# When the Doctor Becomes the Delusion: Fregoli Syndrome Emerging During Resolution of Catatonia Resulting in Therapeutic Rupture

**DOI:** 10.1192/bjo.2026.11885

**Published:** 2026-06-30

**Authors:** Gayathri Rangith

**Affiliations:** Cygnet Health Care, Oldbury, United Kingdom

## Abstract

**Aims:**

Delusional misidentification syndromes are rare but clinically disruptive phenomena. Fregoli syndrome, characterised by the belief that a familiar individual is disguising themselves as others, is particularly uncommon in general adult psychiatry. This case describes a unique presentation in which Fregoli-type delusional misidentification was directed towards the treating clinician, resulting in a profound therapeutic rupture and requiring a deliberate and counter-intuitive reconfiguration of care.

**Methods:**

A middle-aged woman was admitted following police detention under Section 136 of the Mental Health Act and managed under Section 2. She presented with acute catatonia, including mutism, rigidity, marked psychomotor retardation, and self-neglect. Lorazepam was commenced following clinical diagnosis, leading to rapid resolution of catatonic features within 24 hours. As catatonia resolved, florid psychotic symptoms emerged. The patient developed a fixed delusional belief that the treating specialty doctor was her first husband’s first wife’s daughter, disguising herself as a clinician and orchestrating her detention with police involvement. Reassurance was ineffective, and the patient persistently refused to recognise the clinician as a doctor, while engaging appropriately with other medical staff.

**Results:**

The delusional misidentification was consistent with Fregoli-type syndrome and had immediate, observable consequences for care. Direct contact with the identified clinician precipitated marked distress, verbal aggression, refusal to engage, and escalation of paranoia, repeatedly disrupting treatment. A consultant-supported multidisciplinary decision was made for the clinician to withdraw from direct patient contact while continuing to coordinate care indirectly. This decision led to immediate improvement in engagement with the wider team. Persistent psychotic symptoms following catatonia resolution necessitated antipsychotic treatment under Section 3 of the Mental Health Act. Over the course of admission, psychotic symptoms resolved fully, insight returned, and the patient was discharged informally with community follow-up. At discharge, the delusional misidentification had resolved completely, with no recollection of its content.

**Conclusion:**

This case demonstrates that Fregoli-type delusional misidentification can directly undermine therapeutic relationships and compromise treatment delivery. It highlights the importance of recognising clinician-directed delusions and reframing clinician withdrawal, when appropriately supported by the multidisciplinary team, as an active therapeutic intervention rather than a failure of engagement. Awareness of delusional misidentification syndromes is essential to guide flexible, patient-centred care in complex psychosis.

